# Schizophrenia as a Disorder of Social Communication

**DOI:** 10.1155/2012/920485

**Published:** 2012-05-20

**Authors:** Cynthia Gayle Wible

**Affiliations:** Laboratory of Neuroscience, Department of Psychiatry VA Boston Healthcare System, Harvard Medical School, Psychiatry 116A, 940 Belmont Street, Brockton, MA 02301, USA

## Abstract

Evidence is reviewed for the existence of a core system for moment-to-moment social communication that is based on the perception of dynamic gestures and other social perceptual processes in the temporal-parietal occipital junction (TPJ), including the posterior superior temporal sulcus (PSTS) and surrounding regions. Overactivation of these regions may produce the schizophrenic syndrome. The TPJ plays a key role in the perception and production of dynamic social, emotional, and attentional gestures for the self and others. These include dynamic gestures of the body, face, and eyes as well as audiovisual speech and prosody. Many negative symptoms are characterized by deficits in responding within these domains. Several properties of this system have been discovered through single neuron recording, brain stimulation, neuroimaging, and the study of neurological impairment. These properties map onto the schizophrenic syndrome. The representation of dynamic gestures is multimodal (auditory, visual, and tactile), matching the predominant hallucinatory categories in schizophrenia. Inherent in the perceptual signal of gesture representation is a computation of intention, agency, and anticipation or expectancy (for the self and others). The neurons are also tuned or biased to rapidly detect threat-related emotions. I review preliminary evidence that overactivation of this system can result in schizophrenia.

## 1. Introduction

Is there a system for dynamic moment to moment social communication in the brain? Social perception has now been extensively studied in humans and nonhuman primates and is defined as follows: “…the initial states of evaluating the social communicative intentions of others by analysis of eye-gaze direction, facial expressions, body movements, and other types of biological motion.” [1]. Recent evidence suggests that such a system does exist and that abnormal activity in this system may produce the symptoms and cognitive deficits that comprise the syndrome of schizophrenia. A posterior system will be described whose activity may correspond to or underlie the perceptual experience of conversing and interacting with others. Until recently, communication has primarily been studied through language research. This line of research has focused mainly on the structure of the representation and the neural basis of language input (graphemes, phonemes), the internal lexical/semantic representation, and language output (writing and speech production) and has made significant progress in understanding language. 

## 2. Communication Involves Not Only Language Processing but Also Social Cognitive Functions

Communication with another individual involves a set of representations or processes that are outside the scope of traditional language research. Many of these representational systems are studied under the rubric of social cognition. Social communication (in humans) necessitates the dynamic perception of speech, social-emotional cues, and the production of the communicative message (narrative, intonation, and gestures).

**Figure 1:**
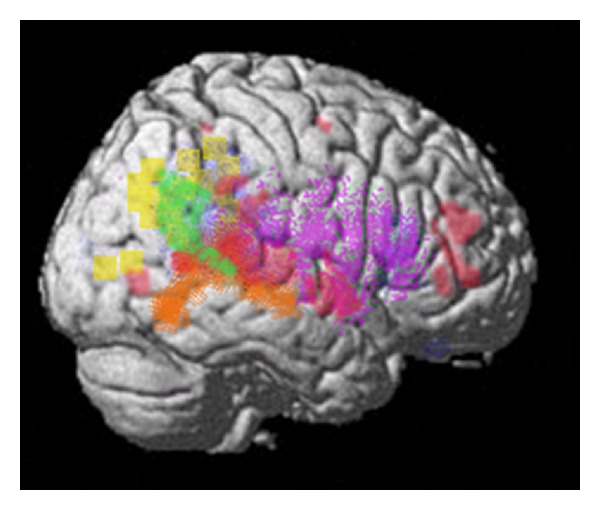
Summary figure of the overlap of functional regions in the TPJ (inferior parietal and PSTS); this region is involved in eye gaze (red); audiovisual speech (orange); self-representation (yellow); theory of mind (green); emotional perception of faces and prosody (dark blue and purple). Rerepresentation of data is, respectively, from [37–40, 47].

**Figure 2:**
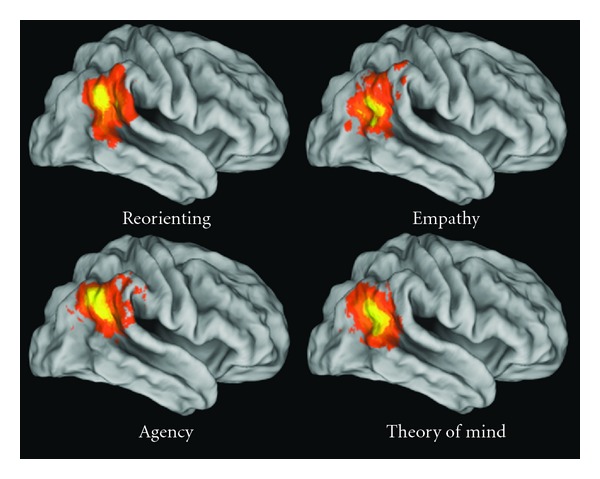
Results of a quantitative meta-analysis of 70 functional neuroimaging studies. Activation likelihood estimation maps in the right temporoparietal junction are shown. Colors code the probability of activation with brighter yellow indicating the highest activation (reprinted with permission from [2]).

The speech signal that is used to communicate is (primarily) both auditory and visual in nature. In naturalistic settings, the perception of speech occurs simultaneously with the dynamic visual perception of gestures, especially of the face. The audiovisual speech signal not only conveys meaning through words but also contains information about emotion (facial expressions and prosody). Perception during conversation also necessitates a host of other functions that are crucial for communication. During the rapid interplay of conversation, the ability to anticipate other's thoughts and actions is also essential. Theory of mind is used; this is the ability to “think about thoughts” and to represent other's point of view. The representation of agency (who is doing what) and intention, both in other's and in one's self, are important. The correct deployment of attention and working memory are needed as well as the ability to follow or track other's behavior and speech. A narrative of the conversation must be built up and maintained; this is an understanding of the context and the story or message that is being conveyed by the individual words and sentences. During conversation, meaning is built up not only from words but also from body and facial gestures as well as intonation (prosody). Recent evidence suggests that there may be a set of brain areas whose functionality matches the representations and processes needed during moment to moment social communication [2–6]. The temporal parietal occipital junction or TPJ (especially in the right hemisphere) is referred to as the “social brain area” [7] because it has been implicated in numerous neuroimaging studies of social cognitive functions such as theory of mind, empathy, social attention, and other functions [2, 3]. Figures 1 and 2 show the functional architecture of the TPJ. It has been hypothesized that the TPJ is involved in lower-level processes associated with empathy, theory of mind, agency, and attentional orienting to salient stimuli and that these processes are crucial to higher-level social function [2] (see Figure 2). The TPJ includes the inferior parietal region as well as the posterior superior temporal sulcus (PSTS) and gyrus (note that only the right hemisphere is shown, but the left TPJ region is also involved in these functions). The functional territory for the PSTS is often thought to extend inferiorly to at least the posterior middle temporal gyrus. The notion that the TPJ is crucial for social interaction/communication has now been tested directly in human subjects. A recent naturalistic functional magnetic resonance imaging (FMRI) study of live face-to-face interaction within an MRI scanner showed strong evidence that the TPJ is a core region for moment to moment social interaction [4]. Medial prefrontal regions have also been hypothesized to play a role in social functioning and data on this point will be reviewed (predominately in the later section of the paper). 

## 3. General Characteristics and Anatomical Connections of the TPJ

The PSTS has a major role in the perception of dynamic gestures. A basic function of the inferior parietal region is in the formation of intentions for action and this region (as well as the intraparietal sulcus) contains mirror representations that are active during either the perception or production of an action [8]. The intraparietal sulcus separates the inferior and superior parietal lobe. Portions of the intraparietal sulcus such as the lateral intraparietal region or LIP are thought to contain a dynamically constructed saliency map that is used to guide gestures such as saccades [8–10]. The TPJ and surrounding regions project to the inferior frontal region and are closely anatomically associated with hippocampal and insular regions [5, 6, 11]. Interoceptive and homeostatic body signals activate the insula (including thirst, sensual touch, sexual arousal, warmth, heartbeat, and bladder distension) [12]. Subjective ratings of these body signals correlate most strongly with activation in the anterior insula and this region has been hypothesized to underlie emotional awareness [12]. Craig describes the anterior insula for “feeling emotion and homeostatic and body feelings” relative to the anterior cingulate's limbic motor function or role in initiating or motivating behavior.

## 4. Social Cognition Is Abnormal in Schizophrenia

It is well known that social cognitive deficits form a major part of the schizophrenic syndrome. Social cognitive deficits are considered to be a major determinant of functional outcome for schizophrenic individuals [13]. Social responsiveness is affected in schizophrenia; the negative symptoms include asociality as well as a lack of facial movement, facial expression, eye contact, and vocal inflection (prosody). In fact the four most prevalent so-called negative symptoms of schizophrenia are a lack of expressive gestures, a lack of vocal inflection and social inattention, as well as a general impairment in attention. Schizophrenic individuals also present with theory of mind deficits and these deficits are related to a lack of expressiveness [14, 15]. It is also important to note that internal experiences of emotion and related sensations (perhaps related to insular function) do not seem to be as abnormal in schizophrenia as emotional responsiveness [16, 17].

## 5. Building Links between the Syndrome of Schizophrenia and Brain Dysfunction: a Parsimonious Account of Positive and Negative Symptoms as Stemming from the Same, Similar, and/or Adjacent Social Cognitive Modules within the TPJ

These negative symptoms and social cognitive deficits could clearly be described as problems with social communicative representations or systems. However, I will argue that most, if not all, of the schizophrenic syndrome can be understood under the rubric of social communication. Recent advances in understanding this system will be described and used to reframe the symptoms of schizophrenia and to then link them to brain function. It is proposed that the overactivation of the functional modules in the TPJ that subserve social communication produces the syndrome of schizophrenia. Direct evidence for TPJ involvement in schizophrenia will be presented near the end of the paper. The formulation presented here is new and remains to be systematically tested. However there are many lines of evidence that will be described below here in after. For example, schizophrenic symptoms are evoked by stimulation to the TPJ [18–21], electrical brain activity within the TPJ coincides with the presence and absence of symptoms [22], and the disruption of TPJ activity alleviates symptoms [23–28].

## 6. Reframing the Schizophrenic Syndrome and Evaluating the Evidence for the Involvement of the TPJ in Social Processing

Understanding aspects of this system at the neural (single cell) and systems neuroscience levels may provide a basis for the understanding the disparate symptoms and cognitive deficits found in the schizophrenic syndrome. Several principles were used to guide the construction of the current framework. Neuroimaging alone cannot provide evidence for a region's involvement in a particular function and taken alone constitutes relatively weak evidence for a link between a function and a brain region. Lesion studies must also show that the function is impaired when the region is damaged. In evaluating human lesion data it is important to analyze each case individually [29]. The extent of involvement of other damaged regions should be taken into account. Data from patients with brain trauma from head injury or with epilepsy should be viewed with caution as these patients often have diffuse damage that may be missed in a radiologic examination. Converging evidence from several sources provides the strongest basis for positing a relationship between brain and function. For example, in interpreting functional imaging data, it is also important that other sources show convergence with the theory, including brain stimulation, transcranial magnetic stimulation (TMS), and data from neuronal recordings in humans or animals. Data from single case studies with circumscribed brain lesions that contradict a specific theoretical position must be accounted for and are a serious indictment of the theoretical position [29]. For each functional domain discussed here in after, I will cite neuroimaging and lesion data to show that the TPJ is a core substrate for the function (as well as data from the other techniques as discussed previously). When single neuron data are described, I will cite evidence showing similar findings from human studies in order to provide explicit links between cellular properties and systems level function (a detailed description of all the studies cited here can be found in [5, 6]).

## 7. The Phenomenology of the Schizophrenic Syndrome Reframed

For brevity, this paper will describe the most prominent symptoms and their relationship to brain function; a detailed account for the other symptoms can be found in [6]. Auditory hallucinations of voices are the most prevalent symptom [30]. Patients have the feeling that someone is actually there and speaking. Auditory hallucinations can also take the form of hearing conversations between voices. Patients will sometimes report that it seems like there is actually another person or persons in their head. Some types of auditory hallucinations may be due to a misattribution of the patient's thoughts to an outside source (abnormal agency attribution for the voice). Visual hallucinations of people in action are the next most prominent category of hallucination [31]. The most common delusions, delusions of persecution and delusions of reference, are characterized by the feeling of being followed, watched, or that people are secretly communicating using gestures or clothes [32]. The prominent positive symptoms of schizophrenia revolve around *the perception or feeling of an entity* that is speaking, communicating, watching, following, observing, or spying. Schizophrenia is also characterized *by abnormal agency judgments*; patients can feel as though their actions and thoughts are controlled by an outside force or entity (e.g., delusions of control, thought insertion, thought broadcasting). Therefore, the prominent hallucinations and delusions may be reframed as the misperception of a social entity or entities or overactivation of representations in the TPJ that subserve many aspects of dynamic social processing. Throughout the paper I will review properties of dynamic social perception and interaction that may be important for understanding the schizophrenic syndrome. For example, recent advances in cognitive neuroscience show that the perception of a social entity is accompanied by an intrinsic and automatic perception of agency, intention, and social prediction [33–36]. These functions are intrinsic to the representation of dynamic action at the single neuron level [33, 34, 36]. It has also been shown that self and other processing overlap and the feeling of a presence can also stem from abnormal activation of the self-representation [19–21, 37]. The prominent negative symptoms are related to abnormal social responding and a lack of social expression or response primarily in the face and voice. The regions that are used to perceive dynamic social interaction are also used in the formation of intentions for the production of social, emotional, and attentional gestures as well as for theory of mind [2, 7, 38–42]. This reframing of the syndrome is parsimonious in that it postulates that the symptoms can be understood as the integrated action of one region (as opposed to widespread abnormalities or complex interactions between anterior and posterior regions).

 In the following sections, I will describe the functional architecture (the arrangement or juxtaposition of functional regions) as well as properties of the neuronal representation of dynamic social action that can be used to understand the schizophrenic syndrome. The functional contributions of the TPJ have been described in several disparate subfields of neuroscience and cognitive neuroscience that are conceptually overlapping but motivated by the investigation of different psychological functions [2, 5, 6]. In describing these findings, I will also show how individual schizophrenic symptoms can be reframed and organized under the rubric of TPJ function.

## 8. The Communicative Signal Is Based on TPJ Function, Is Multimodal (Especially Auditory and Visual), and Carries an Automatic Computation of Agency, Intention, and Social Prediction

The neural code used by the TPJ reflects the fact that communication occurs not only through auditory language comprehension but also through gestures that can occur simultaneously with the speech signal. A basic and prominent function of the TPJ is to represent dynamic gestures, especially in lateral temporal and inferior parietal regions centered on the PSTS in humans [42]. Lesions and repetitive transcranial magnetic stimulation (rTMS) to these regions impair biological motion perception [43, 44]. The homologous regions in the monkey have neurons that are multimodal and respond to the sight, sound, and somatosensory aspects of biological motion [41, 45]. Single-cell recordings in monkeys show that audiovisual representation is the most predominant type of representation followed by visual-tactile representation [45]. The PSTS combines information about biological motion, social/biographical information and speech [41, 46]. In humans, the representation of audiovisual speech occupies a large portion of the TPJ territory (see Figure 1 [47]). These relative proportions match the fact that hallucinations of voices and the feeling that someone is speaking predominate in schizophrenia followed by visual hallucinations of people and then by tactile hallucinations. Hearing voices also activates the TPJ [48]. 

Multimodal gesture representations have properties that allow the neurons to participate in more conceptual functions such as the computation of agency, the ability to keep track of other's behavior, and the ability to predict the actions of others (social prediction) [33, 34, 49–51]. For example, repetition suppression in the monkey STS creates a situation in which neurons that are tuned to sequences of dynamic gestures are maximally responsive to gestures that are about to occur [33]. Single-cell recordings in monkeys have shown that these functions are automatically computed and are an inherent part of the gesture representation at the single neuron level. Neuroimaging has confirmed that activity in the PSTS in humans is also modulated by the perceived intentionality of the gesture or movement [35]. In addition, the human STS was found to be a region in which interactions occur between observed action and the reafferent motor-related copy of that action [52].

Hence, overactivation of this dynamic gesture system could produce the erroneous perception of speech, visual human action (visual hallucinations of people), or tactile hallucinations. Direct evidence for this supposition comes from the fact that cortical stimulation of the TPJ (in nonpsychotic individuals) and lateral posterior temporal lobe can result in visual hallucinations of action scenes involving people [18]. Cortical stimulation of the TPJ can also cause feelings of limbs or body parts changing shape in nonpsychotic individuals (e.g., somatosensory hallucinations and delusions [19]). The overactivation of gesture representations could be graded so that some patients might experience frank hallucinations and others might experience a feeling that there are persons acting, watching, or communicating with them. In addition, this overactivation would be predicted to produce a feeling of agency or of an agent with intentions and would be expected to interfere with social prediction and social interaction.

Activity in these PSTS/TPJ multimodal neurons that encode gestures (especially audiovisual gestures) has been shown in monkeys and humans to provide rapid feedback excitation (within 30 msec) to unimodal regions and to result in gamma synchronization [53]. This phenomenon has been documented in several studies (reviewed in [5, 6]). Hence, erroneously activated audiovisual speech representations may cause the aberrant activation of auditory cortex and the gamma synchronization of TPJ and primary auditory regions. This would result in increased attention and activity in auditory channels.

## 9. The TPJ Is a Core Substrate for Theory of Mind

**Figure 3:**
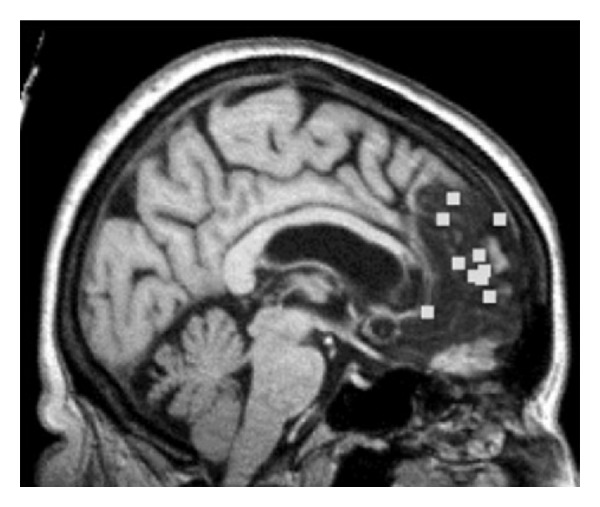
Figure from Bird et al., 2004, showing the lesion site of a patient with anterior cingulate damage who did not show theory of mind impairments. Peak activation foci related to theory of mind from ten different neuroimaging studies are shown (boxes) superimposed onto the lesion site. The figure shows that a lesion in the region of the anterior cingulate activated by theory of mind tasks does not cause impairments on these tasks (reprinted with permission from [57]).

The TPJ is a core neural substrate for theory of mind or the ability to understand and represent another's point of view or thoughts [39, 54, 55]. Neuroimaging studies of theory of mind have shown several foci including the TPJ, the orbital frontal cortex, amygdala, anterior insula, and medial frontal regions [56]. The medial prefrontal cortex or the anterior paracingulate cortex—approximately corresponding to Brodmann area (BA) 9/32—had been thought to be a key region for theory of mind function [56]. However, previous reports of an association between theory of mind, social processing, and the medial prefrontal cortex have relied on patients with traumatic brain injury or epilepsy. These types of patient often have diffuse brain damage that is difficult to detect in radiologic exams (see Bird et al. (2004) [57] for a critical discussion of these studies). There have now been several reports of circumscribed lesions to the medial prefrontal cortex where the patients *do not show theory of mind impairments* [57–60]. Bird et al. (2004) [57] present a carefully documented case of a patient whose injury is circumscribed and coincides with the region identified in several fMRI studies to be involved in theory of mind (see Figure 3). This individual did show impairments in planning and memory as well as a tendency to confabulate but did not show theory of mind deficits on any of a battery of tasks [57]. In another study, three patients with anterior cingulate damage were tested on theory of mind, social situation processing, and motivational decision making. Two of the patients had selective anterior cingulate damage (one also had temporal lobe damage). These two patients were *not impaired in theory of mind, motivational decision making, or social situation processing* [58]. 

TPJ lesions *do impair* theory of mind [60]. In addition, recent neuroimaging studies have shown that the (right) TPJ has a response profile that is more selective for theory of mind than other regions such as the posterior cingulate and the medial prefrontal cortex. The right TPJ was selectively involved in the attribution of mental states rather than reasoning about general socially relevant facts about a person [54]. The ability to make inferences about mental states or beliefs and intentions during moral judgments was also impaired when TMS was applied to the right TPJ [61]. Neurons in the monkey STS that respond to dynamic action or biological motion are tuned to respond to physical or vocal actions that are about to occur or to anticipate action [33, 49]. Aberrant activity in the TPJ would interfere with theory of mind computations resulting in abnormal or slowed social behavior. This abnormal activity would also interfere with dynamic social response and the tracking and hence understanding of other's social behavior. These impairments are core components of the schizophrenic syndrome which includes symptoms such as asociality and social inattention as well as theory of mind deficits as described previously [13–15, 62, 63].

## 10. Emotional Perception and Reaction

Emotional gesture perception and reaction for the face and body as well as prosody perception are dependent on the TPJ, presumably because of its role in voice and gesture representation (e.g., eyes and face). Emotional perception and reaction are linked; the perception of facial expressions produces a subtle version of the expression on the viewer's face [64]. The perceptual circuits that are used to perceive other's actions are also used to represent and plan our own actions, and several regions are active during both observation and imitation of dynamic gestures [7, 64, 65]. Single neurons in the monkey PSTS respond to emotional gestures [49]. Human neuroimaging studies also show that the TPJ is involved in emotional gesture and prosody perception [66, 67]. Lesions within the primary/secondary somatosensory cortex, supramarginal gyrus (part of inferior parietal cortex/TPJ), and insula (adjacent and medial to the TPJ) result in a deficit in the ability to perceive facial emotion and prosody [40]. The supramarginal gyrus may be especially important for the creation of the intentions or plans for emotional and prosodic reaction. Hence, the overexcitation of emotional gesture perceptual circuits could interfere with reaction or with the formation of intentions for facial emotional reaction. This would result in social unreactivity or asociality as well as a lack of facial movement, eye contact, vocal inflection, and facial expression; these are all prominent negative symptoms of schizophrenia. 

## 11. Eye Gestures, Social Attention, and Agency

Movements of the eyes are especially important in social cognition and communication; they not only convey emotion but also convey the focus of attention. Dynamic representations of emotional gesture and attention are used in the perception of more conceptual aspects of social cognition [35, 68]. The TPJ and especially the posterior STS and superior temporal regions are core components of a system for the representation and perception of eye gaze [38, 69, 70]. The TPJ was shown to be selectively involved in representing joint or social attention in a neuroimaging investigation of live social interaction [39]. The perception of gaze direction and the control of attention via gaze are impaired with TPJ damage [70, 71]. Aberrant activity in this system could produce a deficit in the tracking of others eye gestures resulting in abnormal social attention and hence social reaction and understanding.

As discussed previously, the perception of intentionality and agency are an inherent component of the perception of dynamic gestures, including eye gaze [35, 72–74]. For example, in the monkey, neurons in the STS combine information about gestures of the arm (e.g., reaching) with information about the direction of gaze (the focus of attention) to compute agency or intentionality [34]. Overactivation of gaze and other gesture representations would produce the qualia not only of a presence (of being watched) but also of a presence with intentions. This assumption is corroborated with data; direct cortical stimulation of the TPJ can cause the feeling of a “shadowy presence” [20].

## 12. Gesture Neurons Are Tuned to Rapidly Detect Threat

Another property of gesture representation in the TPJ is that the neurons in this neural circuit are tuned to rapidly detect emotionally negative or socially threating gestures. The amygdala is most often cited with regard to this function. However, amygdala damage does not cause a deficit in the detection of dynamic body expressions of fear [75, 76]. This means that there is another brain region that is the core substrate for this function. Single neuron recording in the monkey STS showed that the neurons are tuned or biased to detect potentially threatening biological motion [41]. Event-related potential (ERP) and neuroimaging studies in humans have confirmed that the TPJ is used to perceive threating or emotionally negative gestures [41, 77]. Hence, although this region is generally used to perceive dynamic gestures, there is a neural tuning for the detection of social threat. Hence, overactivation of these gesture representations might produce the feeling of being in a socially threating situation. This feeling could be a component of delusions of persecution and other symptoms.

## 13. Self-Representation, Embodiment, and Agency

The perception of self-made gestures from our own visuospatial perspective and the corresponding auditory, visual, tactile, and vestibular information resulting from actions may provide the concrete scaffolding that is used to create and sustain the self-representation or the feeling of being an embodied, thinking, and acting person [37]. There are also more fixed body schemas that are presumably constructed through experience and that are used in the dynamic representation of the self (these are also located in the vicinity of the TPJ—in the intraparietal sulcus and the secondary somatosensory cortex; see [6] for a summary). Our sense of self is so evident and constant that it may be surprising to learn that it is actually a perceptual construct. However, this construct can break down and be demonstrably associated with certain neural circuits in the same way as other psychological functions such as memory and language. Changes in the feeling of embodiment can be affected by manipulating visual-somatosensory percepts that are represented within the TPJ, by stimulation of the TPJ, or by lesions in the TPJ [37, 78]. For example, the experimental manipulation of visuospatial perspective and simultaneous tactile stimulation can cause the feeling of inhabiting a different body or body swapping [79]. Stimulation or lesion in the TPJ region can cause out-of-body experiences and other changes in the feeling of embodiment [19]. For example, a patient (Susan) with right temporoparietal damage was reported who had a “Capgras syndrome for her mirror image” [80] (see pages 73–75). This patient used sign language and was observed communicating with the person in the mirror (her own reflection). She believed that there was this other “Susan” in the mirror who had the same background, appearance, and age and went to the same school as she did (but she did not know her in school). The other Susan was judged to be a bit slower and not as good at sign language as the real Susan.

The regions and representations that are used to represent the self overlap with those that are used to represent others (see detailed discussion in [6, 37, 81]). The regions that are thought to compute the self-representation are also activated by the perception of dynamic visual (gesture), auditory, or somatosensory stimulation from others, including the TPJ, somatosensory cortex, secondary somatosensory cortex, the extrastriate body area, the intraparietal sulcus, and ventral premotor regions [42, 82]. For example, the secondary somatosensory area responds to touch regardless of whether it was felt on one's own skin or seen on another [83]. Most of the functional components that have been identified with self-representation in the TPJ are modulated by auditory, visual, and tactile inputs and hence overactivation of these channels would be expected to disturb the self-representation [6].

Abnormal feelings of agency and of a presence could also occur as a result of a disturbance of the TPJ self-representation (reviewed in [5, 6]). The bulk of the experimental evidence shows that abnormal activity in TPJ regions and vestibular cortex (insula/inferior parietal lobe) can produce these phenomena [21]. Heautoscopy, the experience of an alternate self or Doppelganger, is associated with either abnormal activity or damage in the TPJ [21]. Visual hallucinations of people are the second most prominent type of hallucination in schizophrenia and may arise from abnormal activation of the self-representation. When the body double is seen (visual hallucination), the visual appearance may or may not mirror the person's own image, but the imitation of body gestures by the double can produce the feeling that the double contains the *real* mind. The experience of the self can be perceived to arise from the body double or can alternate between the physical body and the body double. This type of hallucination can also be experienced as a feeling of a presence or multiple presences of closely projected doubles that are not visible [21]. This feeling of a presence has been induced by electrical stimulation of the PSTS region [20]. Aberrant activation of the self-representation would be expected to interfere with feelings of embodiment and agency for one's thoughts and actions as is seen in symptoms such as delusions of control and could also produce the qualia (feeling of a presence) associated with delusions of persecution, delusions of jealousy, and delusions of reference or visual hallucinations.

Several neuroimaging studies of schizophrenia have shown that abnormal agency processing in patients is related to overactivation in (especially the right) IP or TPJ (reviewed in [5, 84]). For example, one study of agency processing found abnormal activity in the right inferior parietal region in schizophrenic subjects and this overactivation was also positively correlated with the Schneiderian score [85].

## 14. The Medial and Orbital Frontal Regions, Social Cognition, and Self-Representation

Impairments in self-awareness related to abnormal activity in midline structures have been hypothesized to play a role in the social processing problems that have been identified in schizophrenia [86]. Several central and medial regions of the brain, including those in the frontal lobe (medial orbital, the ventromedial prefrontal cortex, the anterior cingulate cortex, dorsomedial prefrontal cortex), have been theorized to be involved in self-referential processing and self-reflection [87, 88]. The evidence for this supposition comes mainly from neuroimaging studies where central midline regions have been found to show activity in tasks such as the recall of personal information, the assessment of self-personality traits, appearance, attitudes, or feelings [87, 88]. Gillihan and Farah (2005) [89], in a critical review of this literature, noted that confounding factors were not adequately controlled in many of these studies. A recent study used TMS to disrupt processing and to probe the brain response to self-related stimuli with non-self-related stimuli. Superior performance on self-related items (the self-reference effect) was found. TMS to left and right parietal cortex suppressed the self reference effect, but no effects on the self-reference effect were found with TMS to the medial prefrontal lobe [90]. It was discussed previously how patients with circumscribed and well-documented medial frontal lesions do not present with a deficit in social situation processing nor have deficits in self-related processing been reported [57, 58, 91]. Confabulation is known to occur with frontal lobe damage [80]. The current framework assumes that delusions are not confabulations but rather explanations of bizarre sensory phenomena as suggested by Brendan Maher (2006) [92].

The dorsal anterior cingulate has previously been associated with action monitoring or the monitoring of response conflict and in cognitive control (see review in [93]). This function is thought to be used in social situations [87, 93, 94]. However, there are now several reports of individuals with circumscribed lesions of this area. These individuals have not been found to have deficits in tasks used to measure response conflict such as the Stroop nor in behavioral measures of cognitive control; see for example, [91]. In fact, recent evidence suggests that the dorsal anterior cingulate response to errors may be more related to levels of negative affect during task performance than to response conflict [95]. These results are consistent with the view that the dorsal anterior cingulate plays a role in the control of autonomic responses that accompany cognitive effort (see review in [91]). The dorsal anterior cingulate may be involved in response selection based on reward contingencies; data from single unit studies in monkeys and in humans and further lesion data from humans undergoing cingulotomy support this hypothesis (see review in [91]). The dorsomedial aspect of the prefrontal cortex may have a role in empathy, in representing emotional pain in others, and/or in the ability to use emotional pain or negative consequences to constrain behavior [96]. Although empathy and theory of mind scores are correlated, the precise relationship between them is unclear [57]. A recent FMRI study found that the anterior middle cingulate cortex responded to both emotionally and physically painful events, while the dorsomedial prefrontal cortex responded selectively to emotional pain [96]. A lack of empathy does not seem to be central to schizophrenia, although it is a symptom of the manic phase of bipolar disorder.

Orbital and ventral medial frontal lesions also do not cause theory of mind deficits [97] but rather result in an acquired sociopathy [98]. Orbital and ventral medial frontal lobe damage has generally been reported to result in a lack of empathy, euphoria, irresponsibility, a lack of concern for the future, and a lack of concern for social rules [97–99]. When tested explicitly on measures of social judgment and responding, individuals with orbital and inferior medial frontal lesions present with an intact knowledge of social rules and the ability to judge the outcomes [98]. However, their autonomic responses to socially meaningful stimuli may be abnormal and it is has been hypothesized that these patients fail to activate somatic (body emotional) states in response to social stimuli [99]. Another report surmised that orbital damage results in a reduced ability to use the expectation of negative emotional reactions (anger) to constrain behavior [97]. Recently it has been reported that orbitofrontal lesions cause a diminished sensitivity to varying reward magnitudes [100]. These data are consistent with single unit recordings in monkeys showing neuronal responses that were associated with various aspects of reward computed from experience with behavioral choices [101]. It is claimed that damage to the orbital and ventral medial prefrontal cortex leads to an inability to “develop a coherent model of one's own self” and hence emotional liability and aberrant social functioning [87]. However, the function of orbital and ventral medial frontal cortices has been repeatedly shown to be related to an inability to use negative emotions to constrain behavior or to competently compute and use reward or punishment outcomes. This syndrome seems more closely aligned with mania where decision making is impaired and individuals often do risky things without regard to the emotions of others (manic symptoms include euphoria, lack of empathy, impulsiveness, lack of concern for consequences of behavior and for social rules) and manic patients sometimes confabulate as is seen in patients with frontal lobe lesions [80].

In summary, medial prefrontal regions have been shown to be activated during tasks assessing theory of mind and self related processing; this presupposes a role for these regions in social processing. Anterior cingulate regions show coactivation with the TPJ in many neuroimaging studies and hence abnormal activity in the TPJ could be expected to affect activity in this region. However, as yet there are many competing theories concerning the role of this part of the system and hence it is unclear what the functional consequences of abnormal activity would be and what form they would take (overactivation, lack of activation). 

## 15. Direct Evidence for TPJ Involvement in Schizophrenia

It is hypothesized that the TPJ (and primary efferent areas) should show slightly elevated baseline activity interposed with epochs of hyperactivation that correspond to the abnormal qualia (hallucinations and delusions) and responsive deficiencies (negative symptoms) seen in schizophrenia [5, 6, 102]. Although it is not often acknowledged (perhaps because of the almost axiomatic belief in frontal lobe abnormalities), there is strong evidence for TPJ involvement in psychosis and schizophrenia. Activity has been recorded in the TPJ that corresponds to the experience of symptoms, activity has been found that is correlated with symptoms, and recent large-scale morphometric studies show volume reductions in the TPJ in schizophrenia. Torrey [103] wrote an extensive review of the evidence for inferior parietal involvement in schizophrenia that includes an account of the relationship between prodromal symptoms and parietal function (we will not reiterate that evidence here). The TPJ region is a relatively anatomically variable region and hence volume abnormalities could be relatively difficult to detect; the inferior parietal region is one of the last regions in the brain to be myelinated and develops into late adolescence [104]. However, recently two large-scale morphometric studies of schizophrenic volume reductions have been published. Both of these studies show core reductions in TPJ volumes [105, 106]. Direct evidence for abnormal activity in the TPJ during psychosis comes from brain recordings; right inferior parietal activity was related a delusional state in a magnetoencephalography (MEG) study [22]. When the abnormal activity subsided, the delusions subsided. Correspondingly, interference with TPJ activity alleviates schizophrenic symptoms. At first TMS was applied to the TPJ to treat auditory hallucinations; at least 6 published studies have now shown that TMS applied to the TPJ alleviates auditory hallucinations and some reports show reductions in other symptoms as well [23–28]. For example, in one study schizophrenic subjects with treatment-resistant auditory hallucinations were given daily rTMS treatments for 10 days and showed a reduction in the frequency of auditory hallucinations and an improvement in many other symptoms [23].

Data from our previously published studies have consistently supported TPJ involvement; schizophrenic symptoms such as auditory hallucinations and thought disorder were correlated with levels of FMRI activity in the inferior parietal and superior temporal sulcus in several studies [107–109]. One of the most systematic FMRI symptom capture studies of schizophrenia was one of a single schizophrenic subject who heard voices for approximately 26 seconds and then no voices for approximately 26 seconds; this periodicity is relatively optimal for an FMRI design [110]. FMRI activation was detected surrounding the PSTS approximately 3 seconds prior to the conscious detection of the auditory hallucination. Activation of the inferior parietal and inferior frontal regions followed. The PSTS activation (which extended into the superior and middle temporal regions) was persistent through the entire experience. This coincides with the unusual extended neural responses (e.g., 7 seconds) recorded in monkey STS neurons that are activated by dynamic multimodal gestures [49].

TPJ over-activation could stem from a number of causes, but it is hypothesized that in schizophrenia the source is most often from over-activation of the hippocampal system [111]. The activity of the hippocampus shows a high association or correlation with activity in the TPJ (so-called default mode or cortical hub activity) and evidence from epileptic individuals shows that abnormal hippocampal activity underlies active psychosis and that the TPJ region is also involved [6]. Note that resting state abnormalities have been repeatedly found in schizophrenia and that a seed placed within the hippocampus will produce activity in most of the “default mode” regions [112–115] (see [6] for a detailed discussion of hippocampal involvement).

## 16. Summary

In summary, the regions that make up the TPJ form a core system for the perception and production of emotional face and body gestures as well as prosody. This region shows functional activity that indicates that it is preferentially involved in the perception of narrative (versus words or sentences) [3]. This area is a core component for the perception of social attention or of the process of joint attention (the deployment of attention according to social and communicative cues). This area is also the core region in the brain for theory of mind or understanding other's intentions and thoughts. The neural activity in this system is biased to detect or anticipate future multimodal social acts, or in other words to anticipate speech and actions. Inherent in the representation of multimodal gestures is a coding of intention and agency. Even though this system is used for the perception and production of biological motion or gestures, there is a neural bias or tuning for the rapid identification of social threat and parts of the TPJ are core components of fear perception (reviewed in [5, 6]). The TPJ not only represents other's actions but may also be a core area for representing the self. This is the only region of the brain that produces verbal memory deficits when damaged and hence is the neural substrate for verbal working memory (reviewed in [5, 6]). Since neural activity in this region corresponds to social perception and joint attention, over-activation of this region could result in the erroneous perception of being within a dynamic social situation. Over-activation of this region would have widespread consequences for many domains including social perception, social reactivity, and attention.

When the consistency and weight of the evidence is considered, the characteristics of TPJ function more closely match the symptoms of schizophrenia. Hippocampal activity has been consistently shown to be abnormal in schizophrenia which is highly correlated with both TPJ and anterior cingulate/paracingulate activity [113–115]. The characteristics of medial prefrontal function (especially from the lesion data) may match those of bipolar disorder more closely than those of schizophrenia.
